# Promoting Psychosocial Well-Being and Empowerment of Immigrant Women: A Systematic Review of Interventions

**DOI:** 10.3390/bs13070579

**Published:** 2023-07-12

**Authors:** Patricia Silva, Henrique Pereira

**Affiliations:** 1 Department of Psychology and Education, Faculty of Social and Human Sciences, University of Beira Interior, Pólo IV, 6200-209 Covilhã, Portugal; hpereira@ubi.pt; 2 Research Centre in Sports Sciences, Health Sciences and Human Development (CIDESD), 5001-801 Vila Real, Portugal

**Keywords:** immigrant women, intervention, empowerment, psychosocial well-being, systematic review of literature

## Abstract

This systematic review (SLR), based on the PRISMA 2020 guidelines, aims to present a current overview of interventions aimed at promoting the psychosocial well-being and/or empowerment (PWE) of immigrant women in order to guide future projects. Data collection was performed in the SCOPUS and Web of Science databases, with studies published between 2012 and 20 March 2023 in English, Portuguese, and Spanish. Inclusion and exclusion criteria were based on the PICO guidelines: (P) immigrant women, (I) interventions to improve PWE, (C) comparison between the initial and final phases, and (O) evaluated results for PWE. Risk of bias was assessed, and most of the studies met more than 80% of the JBI bias criteria and had moderate quality on GRADE. Thirteen studies with 585 participants were included, mostly non-randomized, non-equivalent, and with an experimental-control group design. The main components of interventions were health education/psychoeducation, counseling, cognitive restructuring, and expressive therapies. A descriptive synthesis of qualitative and quantitative data was made to evaluate the results of the interventions in PWE. In the experimental studies, results assessed improvements mainly in mood and depression levels, and stress reduction. Empowerment components were less covered. Experimental groups performed better in almost all variables in the comparison with control groups. The strongest interventions were psychoeducation and cognitive restructuring techniques. The main limitations of the studies were the lack of quality of several studies, sample size and representativeness, language, and the possibility of response bias. Even taking this into account, this article makes an original contribution by advocating for evidence-based practice and offering significant implications for health professionals, policy makers, and researchers that work with the integrative health of immigrant women. This SLR is registered in PROPESRO Registration: CRD42023399683. PS is a research fellow of the Foundation for Science and Technology (FCT) of Portugal.

## 1. Introduction

Continuous advances in women’s rights and achievements have been taking place, however, significant inequalities between men and women persist in several sectors, including the labor market, education, and the political environment. These inequalities can manifest themselves through reduced opportunities, pay gaps for equal or similar work, limited access to educational and cultural resources, unequal distribution of domestic and family care work, and denial of fundamental rights [1,2,3,4]. 

Immigrant women experience additional challenges regarding gender inequalities due to the intersection with other issues, such as ethnicity, culture, religion, beliefs, and the economic and social norms of their countries of birth [5]. These women handle these different challenges while they must also adjust to the new environment, often away from their social and family support system, and while being exposed to several vulnerability factors, including poverty, unemployment, discrimination, xenophobia, racism, and situations of violence [6,7,8]. Language barriers are also a critical factor that significantly influence immigrant women’s experiences. These barriers can lead to a range of difficulties, such as limiting access to information, services, and resources, including health care, education, and employment opportunities [9]. Remaining in these unfavorable conditions can result in an increase in isolation and stress and decreased levels of self-esteem, which consequently may lead to poorer psychosocial well-being for immigrant women and negatively affect their empowerment process.

Psychosocial well-being is a complex construct that encompasses variables such as emotional, spiritual, cognitive, behavioral, psychological, and social aspects [10,11]. It includes feelings of satisfaction with life, high self-esteem, effective coping, resilience, a sense of belonging, and social support [10,12,13,14]. However, risk factors such as communication difficulties, constant stress, anxiety, discrimination, social isolation, exploitation, abuse, and traumatic experiences can damage this state [15,16,17,18,19]. Studies have indicated that immigrant women scored worse on assessments of mental health and psychosocial well-being when compared to non-immigrant women [20,21], especially if there is an intersection with other aspects such as the color of the person [22]. 

Empowerment, from a female perspective, is a process in which women are able to take control of their own lives and to be more autonomous and independent [23,24,25]. For immigrant women, empowerment can involve accessing resources and skills to overcome inequalities and vulnerabilities, as well as actively participating in making decisions that affect their lives, including their careers, bodies, relationships, and rights [15,26,27]. However, empowerment can be difficult due to lack of access to resources and opportunities, prejudice, and discrimination [9,15,28]. Furthermore, language proficiency is often associated with social acceptance and inclusion. Without the ability to communicate effectively, immigrant women may face further social exclusion, which can exacerbate feelings of isolation and disempowerment [9]. In this sense, it is essential to implement actions aimed at promoting the PWE of women, especially those who are in a more vulnerable situation, such as immigrant women. These actions can decrease gender disparity and increase women’s participation in society and in the world [29,30,31]. However, despite relevant research in this field, there are few review studies to enable a comparison between studies and provide a baseline for future research. Hence, this study aims to develop an SLR to centralize current knowledge on research that has used interventions to promote PWE in immigrant women by comparing pre-post intervention outcomes. It is hoped that this SLR will present an update of the theoretical framework of interventions, methods, outcomes, benefits, and limitations. Consequently, this study can be a useful guide for public policies and future strategies for this population. It also can be used as a starting point for further research in this field.

## 2. Materials and Methods

This SLR was conducted following the guidelines of the PRISMA 2020 statement: an updated guideline for reporting systematic reviews [32]. The PRISMA checklist was also completed for the abstract (Appendix A) and for the completed SLR (Appendix B). 

### 2.1. Data Collection Strategies

Data collection was carried out through the complete collection of Scopus and Web of Science, and studies included were published from 2010 until the final data collection date, on 24 March 2023. We chose the Scopus and Web of Science databases for our research because: (1) the multidisciplinary and comprehensive scope of these databases allows for research in different areas of knowledge that are related to our topic, such as psychology, sociology, migration studies, public health, and others; and (2) both databases are recognized for their rigorous selection of scientific journals, maintaining a robust editorial selection process to include only high-quality studies. Consequently, they provide reliable and relevant studies. On 25 March 2023, a snowball literature search was conducted to locate other eligible studies for our investigation. This approach is analogous to the “snowball sampling” used in primary research. In our search, we started with the identified body of articles in the databases. Then, the reference lists of these articles were analyzed to find additional studies. This method can help discover studies that the database search may have missed. Ultimately, it aims to reduce selection bias and identify a larger number of relevant studies on a topic. 

After initial research and with the support of the Virtual Health Library platform, the search strategy included terms (and synonyms) related to immigration, women, intervention, psychosocial well-being, and empowerment, in English, Portuguese, and Spanish languages. Given the nuances of these terms in the different languages, Table 1 presents the translation for each of them.

The decision to choose English, Portuguese, and Spanish languages for the studies included was guided by some considerations. These languages are considered among the most spoken worldwide and are particularly prevalent in countries with high rates of immigration. This fact increases the probability of finding studies on the topic. In addition, our team is proficient in these languages, which allowed us to interpret and analyze the studies more precisely, without the risk of misinterpretation due to language barriers. 

The advanced search capabilities of the databases were utilized to ensure successful extraction of results. The keywords from the 3 languages were combined using search tools such as TS field (topic), Boolean operators AND, OR, and the wildcard character * (asterisk) in both databases. Articles that did not fulfill the inclusion criteria, as well as review articles and unpublished studies, were removed from the results. To ensure the efficiency of the search strategy, a validation process was conducted, resulting in the identification of four relevant studies. Appendix C provides more information on this process.

### 2.2. Criteria for Inclusion and Exclusion of Studies

For the inclusion and exclusion criteria we employed the PICO strategy: 

Participants: The population was composed of immigrant women over 18 years old living in a foreign country. Studies conducted exclusively with immigrant women under 18 or over 70 years old, pregnant, or postpartum women, and individuals with intellectual or physical disabilities and refugees were excluded. 

Interventions: We included all interventions specifically designed to improve PWE. However, they were only included if they were clearly identified and protocolled. We classified interventions as “clearly identified” based on 2 criteria. (1) The intervention had to be explicitly stated and described in the study. This includes defining the type of intervention (e.g., counseling, psychoeducation). (2) The intervention had to be specifically targeted to improve PWE. Studies where the intervention was not the primary focus, or the goal was not PWE were not included. Interventions that essentially addressed physical conditions, substance abuse, parenting, or gender/domestic violence were also excluded. We classified interventions as “protocolized” when the program was implemented based on a pre-established structure (set of sessions, database with defined content, or completion of phases). The techniques used, duration, and frequency of the intervention were also to be presented. Therapies that did not have a defined plan were excluded from the study.

Comparisons: Results had to present a comparison between the initial and final phases of the intervention through a quantitative design (controlled trials, quasi-experimental design, and pre-test/post-test studies) or a mixed design (quantitative and qualitative methodology), if they evaluated the effectiveness of the intervention. Studies that did not present a comparison between the initial and final phases were excluded. 

Outcomes: Results had to promote the PWE of the participants. Studies contained measures and analyses that made it possible to verify significant improvement in the empowerment and psychosocial well-being in the population assisted. Interventions whose measures and analyses were not clearly reported were excluded. We classified “measures and analyses clearly reported” when the measures and analysis tools were described in a precise and understandable way, allowing the analysis of our study variable (PWE) and the possibility of study replication. Studies that did not have at least one measure and data analysis tool clearly described were not included.

### 2.3. Article Selection Process

**Figure 1:**
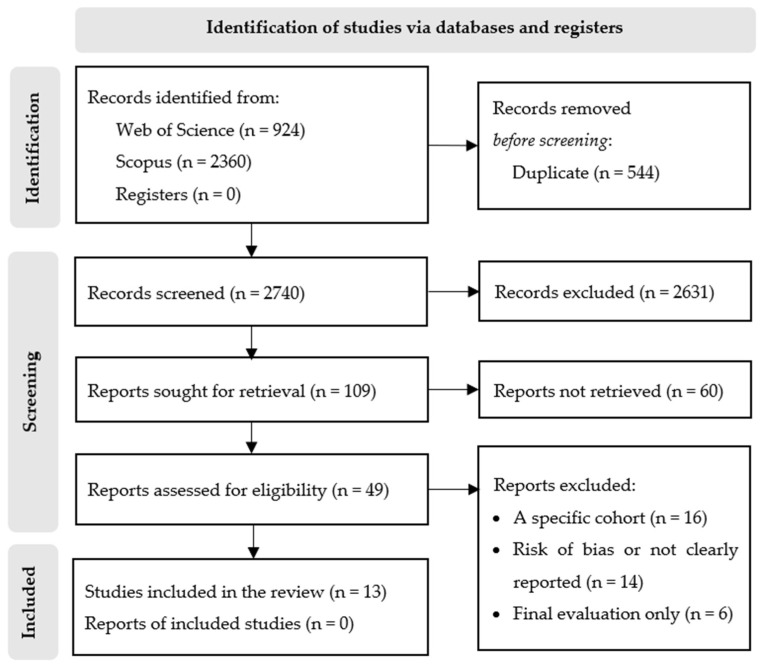
PRISMA flowchart of article search and selection.

Data analysis was conducted using the Endnote Online. Results from databases were saved by title and abstract. Prior to assessing eligibility for inclusion, duplicate citations were removed and archived, first using the Endnote and then manually. Titles and abstracts were reviewed, and studies that were potentially relevant were included for full-text review. Full-text articles were reviewed. A PRISMA flow diagram was completed to describe the number of studies identified. A total of 3284 studies were initially retrieved. After removing duplicates using the Endnote (337) and manual screening (207), the titles and abstracts of 2740 articles were assessed for eligibility based on the PICO criteria, and 2631 were excluded. The main reasons for exclusion were that the target population was not immigrants, women, or they were children, pregnant women, or refugees; or the interventions were aimed at treating physical conditions such as cancer (mainly colon and breast), obesity, cardiovascular disease, diabetes, HIV, gynecological and obstetric issues, vaccination, parenting issues, or substance abuse. The remaining 109 articles were included for full-text analyses, and 60 were excluded. The reasons for exclusion at this stage were mainly because the population was mixed (male and female immigrants, whole families, immigrants, and non-immigrants), or the intervention did not aim for empowerment or psychosocial well-being, or this was not validated. Finally, 36 studies appeared to meet the inclusion criteria, but 20 were excluded because they only had one final evaluation measure or scored very low in the assessment of the risk of bias or did not clearly report the results. A total of 16 studies were excluded because they were implemented in very specific populations, such as caregivers, farm workers, or survivors of domestic violence. Finally, thirteen publications were included in this review. We searched the reference lists of included articles but did not identify any additional articles that met the inclusion criteria. Figure 1 presents the process of selection and inclusion of studies in each phase.

After the studies were selected, we extracted variables such as authors, year of publication, country of study, study design, sample cohort, intervention/program characteristics, data collection and analysis tools used, main results in the theme, and limitations. To prepare the data for synthesis, we categorized the collected variables in an Excel sheet and shared it among the authors. Table 2 presents a summary of the 13 included studies, including the N assigned to each study, author, year, country of study, study design, type of intervention and measures, study participants, and the effect of intervention on PWE.

### 2.4. Descriptive Synthesis of the Studies

#### 2.4.1. Design of Studies

The studies were published between 2012 and 2021, conducted in the USA (N = 6), Republic of Korea (N = 4), and Spain (N = 3). All studies had a pre/post intervention design (N = 13), and three had a follow-up after two [44], four [34], and six [38] weeks. Only one study [44] was a randomized and controlled experiment with a double-blind process. Twelve studies were quasi-experiments, divided into experimental and control group [34,35,37,39,40,44,45], experimental group in two [41] or four cohorts [36], or only a pre/post intervention experimental group [33,38,42,43]. Most studies were non-randomized (N = 7) and/or non-equivalent (N = 6). Only 3 were randomized [40,42,45].

#### 2.4.2. Characteristics of Participants

A total of 584 immigrant women were evaluated and 433 received some type of intervention for the improvement of their psychosocial well-being and/or empowerment. A total of 151 participated in control groups. Sample sizes of the studies ranged from 11 to 75 participants. Seven studies reported that participants should be over 18 years old, and in three studies, over 21 years old. Some of the main inclusion criteria were depression (5), being married to a Korean or being in an international marriage (4), or needing some type of public or financial assistance (4). Women were mostly born in countries in Latin America (32%), Asia (21%), Europe (11%), and Morocco (16%).

#### 2.4.3. Characteristics of Interventions

Our data categorization resulted in 4 main components of the interventions: the study provided counseling in various aspects of life, such as relationships [35], promotion of physical, social, and mental health [36,37,38], migration journey [35,43]; health education/psychoeducation [33,35,41,42,43,45]; cognitive therapies with cognitive reconstruction techniques [33,34,40,41,45]; and behavioral activation [41]. Expressive therapies such as logo-autobiography [34], sandplay therapy [39], and laughter therapy [44] were also implemented. Five studies were conducted through computer or telephone resources [36,37,38,41,42]. Activities developed in a group setting [33,40,43,44], individually [35,36,37,38,42], or both [34,39,41,45] were also mentioned. Studies had an average of 8 sessions, except for studies [36] and [37], which had a face-to-face session and sent text messages for 26 days. The shortest intervention was from [38], which consisted of one week of transmedia engagement via telephone or internet. The longest intervention was from [45], which implemented 12 sessions, every 15 days for 26 weeks. The program content was structured through sessions planned [33,34,35,39,42,43], overall content [36,37,38,41,44], or phases developed for interventions [36,37]. 

We also examined the language used in the implementation of the intervention, studies [33,39,45] did not provide information on the language in which the programs were conducted. Studies [34,35,41,43] reported that the programs were implemented in the native language of the participants. In [36], the program was implemented in the native language of most of the participants, but it is unclear whether all women spoke the language of the program. Studies [37,38,40,42,44] indicated that the programs were conducted in the language of the country, and that the target population was able to communicate in that language. In [44], if the participants did not speak the language, a translator was present to provide translation services. Appendix D provides a description of the interventions in each study.

#### 2.4.4. Instruments for Data Collection and Analysis

The most used instruments for data collection were the Patient Health Questionnaire (N = 6), followed by the Center for Epidemiologic Studies Depression (N = 4), the Final Questionnaire on Text Messages (N = 2), and the Perceived Stress Scale (N = 2). Other tests were used only once (N = 33). Descriptive analyses and sample identification were performed in all studies. The T-test was the most used test for data analyses (N = 8), followed by ANOVA [33,34,40,44], Wilcoxon [33,36,39], Mann–Whitney U test [34,39], and Fisher’s exact test [40,44]. Other analyses were performed only once (N = 12). Semi-structured interviews and qualitative analyses were conducted in 3 studies [41,42,45]. Only study [44] analyzed physiological data (saliva). [33] performed the most statistical analyses (N = 7), while [37] performed the fewest (N = 1). Appendix E provides a description of instruments for data collection and analysis in each study.

### 2.5. Quality of Studies

The risk of bias in the included studies was evaluated using the Joanna Briggs Institute (JBI) Appraisal Checklist Tools [46]. The JBI Appraisal Checklist for Randomized Controlled Trials, consisting of 13 items was used for the randomized controlled trial (Study [45]). For non-randomized studies, the JBI Appraisal Checklist for Quasi-Experimental Studies, consisting of 9 items was used (all other studies). In our investigation, only those studies that fulfilled over 50% of the established requirements were included. The choice of this cutoff by our team allowed us to find a balance between including the studies and ensuring an acceptable level of methodological quality and rigor in our analysis. Quasi-experimental studies had to meet 5 or more of the 9 criteria in the JBI Checklist for quasi-experimental studies. Experimental studies had to meet 7 or more of the 13 criteria in the JBI Checklist for experimental studies. Twelve studies were evaluated and met five [41], six [36,38], seven [33,35,37,42,43,45], eight [39], and nine [34,40] requirements of the criteria established by the quasi-experimental checklist (79%). Study [44] was evaluated using the experimental checklist and met 10 of the 13 requirements (77%). Table 3 shows the requirements for each study and their respective scores.

To evaluate the quality of the studies, we also assessed factors that could potentially interfere with the performance of the tests, such as the provision of payments in exchange for participation in the study. Our analysis revealed that four studies did not address this issue [39,40,43,45], while 3 studies explicitly stated that participation was entirely voluntary and no remuneration was provided [33,34,44]. Six studies offered some type of compensation, such as a gift or a gift card [35,38,41], expenses for phone and internet [36,37], or a financial compensation [42]. We also identified several limitations that may have impacted the reliability and validity of the findings. Some of the main limitations we identified were related to the sample size and/or representativeness [33,35,37,38,40,41,43,44], the possibility of response bias [34,38,41,42,43,44], the lack of a control group [33,36,41,42], the study being conducted with convenience samples [33,41,43], language issues [34,40], and data collection being self-reported [40,42,43]. One study did not report limitations [39]. [45] only pointed out real-life economic constraints when implementing the program as a limitation. In seven studies, the risk of bias was unclear.

Finally, we conducted a Grading of Recommendations Assessment, Development and Evaluation (GRADE) to assess the level of certainty related to the evidence and identify factors that could contribute to bias in the evidence. For this purpose, we defined a Likert scale ranging from 1 to 4 points, and the level of certainty of the evidence from each study was evaluated as high, moderate, low, or very low. Reasons for downgrading the evidence included limitations in study design, sample size and configuration, bias checklist score, quality of data collection and analysis, and reporting of results. Studies with very low quality were not included. Studies [33,34,35,36,37,40,42,43,45] were rated as moderate quality, while [38,39,41] were rated as low quality. Only study [44] was evaluated as high quality.

### 2.6. Data Analyses to Access PWE

Our analyses aimed to summarize the general effects of the implementation of the interventions on PWE and to verify the most suitable approaches for improving the PWE of immigrant women. To facilitate the identification of results, we defined 2 topics for each synthesis we would conduct. (1) For the results of the interventions on the PWE of immigrant women, studies were categorized into: (a) promotion of psychosocial well-being, (b) promotion of empowerment of women, and (c) promotion of both PWE. (2) For the evaluation of the strongest interventions affecting PWE in immigrant women, we performed a score compilation of the best findings for: (a) results in PWE, (b) quality of studies, and (c) descriptive synthesis of the studies. Using these codifications, we were able to identify the studies that met the eligibility criteria for each of our planned syntheses, as well as put them into the categories they best fit, creating a basis for our results. 

Due to the heterogeneity in study designs and outcome measures, conducting a meta-analysis was not possible. Instead, we conducted a descriptive synthesis to summarize the PWE findings of the included studies. The synthesis involved a convergent analysis of the quantitative data, combining and interpreting the results to derive meaningful insights. When statistical aggregation was not possible, results were presented in a narrative format. Our analysis also included a thematic synthesis of the included studies that used qualitative analyses as part of their methodology (N = 2). Each qualitative result was reviewed, and the relevant data pertaining to our theme (PWE in immigrant women) were extracted. Subsequently, these data were coded and grouped into themes for presentation. After the categorization and analyses, the results were presented in a descriptive format.

The entire process of data collection, analysis, and risk of bias assessment was conducted in two stages: Stage 1 involved one reviewer (PS) who examined the studies and extracted the predefined variables. At Stage 2, a second reviewer (HP) verified the extracted data and supplemented them as necessary. Any discrepancies and uncertainties regarding the inclusion of a study, the data analysis process, or the bias categorization were resolved through discussion between the two reviewers (PS and HP). Whenever necessary, a third researcher and expert in the field was consulted for the final decision.

## 3. Results

### 3.1. Results of the Interventions on PWE of Immigrant Women

In our research, we adopted a comprehensive understanding of psychosocial well-being, which encompasses various aspects such as mental health balance, quality of life, and social interactions. Empowerment refers to the dynamic process in which individuals or groups achieve and maintain autonomy, independence, and social participation. This implies, for example, being able to manage their own lives and make their own decisions. In this topic, we evaluated the effects of the interventions on these components across studies with experimental groups and studies with experimental and control groups.

#### 3.1.1. Results of Experimental Studies on PWE of Immigrant Women

For studies conducted with only experimental groups, the main results on psychosocial well-being are observed through a significant difference post-intervention in the improvement of mood and depression levels [36,38,41,43]. There were also significant and positive differences in reducing stress [33,42,43], anxiety [38], and in post-traumatic stress [41]. In the evaluation of empowerment components, significant improvements were observed in increased self-esteem [33], self-efficacy, general empowerment, and safety-related empowerment [42], active coping, positive reframing, self-distraction, and planning [43]. In [43], these results were significantly associated with reduced stress and depressive symptoms. However, Ref. [33] did not show any significant improvements in sense of coherence and mental quality of life. In [36], only statistical differences occurred for experimental groups that showed moderate or high depression levels in the initial evaluation phase. In [38], the follow-up after 6 weeks did not show significant results. Three studies that evaluated variables related to social support did not present significant results after the intervention [33,41,43]. However, in [41], the qualitative analysis showed an improvement in the perception of social support. Except for [38,41], all these studies were rated with moderate quality in the GRADE evaluation and scored between five and seven points on the JBI Checklist for risk of bias for quasi-experimental studies.

#### 3.1.2. Results of Experimental and Control Studies on PWE of Immigrant Women

Studies conducted with experimental and control groups showed significant differences after the intervention, with the experimental group scoring better in almost all variables. In terms of psychosocial well-being, there were improvements in mood and depression levels [34,35,37,40,44,45], anxiety [39,44], stress [45], acculturative stress [35,44], general health [35], loneliness [35,39], and a decrease in salivary cortisol [44]. Regarding the empowerment components, significant improvements were observed in health literacy [35], self-esteem [40], and life purpose [34]. In [34], these scores were maintained at both post-test and 4-week follow-up. However, there were no significant differences between the control and experimental groups in [39] scores, the overall effects of social problem solving [40], and salivary IgA [44]. Qualitative analyses revealed perceived improvements in loneliness, cognitive reconstruction, and financial assets. Except for [39], which rated as low quality, and [44], which rated as high quality, all of these studies were rated as moderate quality in the GRADE evaluation and scored between seven and nine points on the JBI Checklist for risk of bias for quasi-experimental studies. Ref. [44] scored 10 out of 13 points for the JBI checklist for experimental studies.

### 3.2. Strongest Interventions to PWE in Immigrant Women

Based on our findings, we assessed which interventions seemed to be the most effective for promoting PWE in immigrant women. We identified the techniques and approaches used in the included studies and selected those that had the strongest evidence base. The two interventions highlighted for their effectiveness and quality were psychoeducation and cognitive restructuring techniques. 

Psychoeducation was implemented for different topics in six of the 13 included studies [33,35,41,42,43,45]. In [33,35,45], topics such as acculturative stress, stress management, satisfactory relationships with husband, children and husband’s family, mental health literacy, and utilization of mental health services were addressed. Ref. [42] provided individual feedback based on identified strengths and offered educational modules and sessions for stress reduction. In [41], psychoeducation was used as part of the content included in the behavioral activation program. In [43], sessions were provided related to the migration journey, adjustment to a new location, mental health, and social and family issues. These interventions were significantly effective in improving psychosocial well-being related to improvement of mood and depression levels, general health, in reducing stress, acculturative stress, post-traumatic stress, and loneliness. For the empowerment components, the benefits were related to increased self-esteem, self-efficacy, health literacy, general empowerment, safety-related empowerment, active coping, positive reframing, self-distraction, and planning. Except for [41], all of these studies were rated moderate in quality in the GRADE evaluation and scored seven on the JBI Checklist for risk of bias for quasi-experimental studies.

Cognitive restructuring techniques were used in five of the 13 studies [33,34,40,41,45]. Refs. [33,34] implemented sessions that involved producing a personal timeline, exploring relationships before and after migration, and exploring personal crises and attitudes toward those crises. Ref. [40] focused on cognitive reconstruction to promote a positive self-image, and develop personal strengths as well as problem solving skills. Ref. [41] adopted behavioral activation for the identification of problem behaviors and idleness and implemented interventions to increase activity and participation in pleasurable activities. Ref. [45], in turn, focused on developing psychological skills through cognitive strategies to identify symptoms of tension and their associated thoughts and feelings, in addition to strengthening social networks and building friendships. These interventions were significantly effective in improving psychosocial well-being in terms of reducing mood and depression levels, in reducing stress and post-traumatic stress, and loneliness. For the empowerment components, the benefits were related to increased self-esteem and life purpose. Furthermore, Ref. [34] implemented this technique and demonstrated better results on PWE measurements at the 4-week follow-up. Except for [41], all of these studies were rated with moderate quality in the GRADE evaluation and scored between seven and nine points on the JBI Checklist for risk of bias for quasi-experimental studies.

Nonetheless, although the qualitative analysis of [41] revealed an improvement in perceived social support, the studies that used psychoeducation and/or cognitive restructuring techniques for support issues and social problems [33,41,43] did not find significant quantitative results. 

## 4. Discussion

### 4.1. Effects of Interventions on PWE of Immigrant Women

All studies included in the review reported significant improvements in different aspects of psychosocial well-being. Immigrant women who participated in interventions showed improvements in mood and overall health, reduction of symptoms of depression, stress, acculturative stress, anxiety, PTSD, and loneliness. These effects were generally maintained when compared with the experimental and control groups, with a few exceptions. These findings are consistent with other SLRs that investigated interventions to improve the psychosocial well-being of immigrant people using psychosocial variables such as reduction of stress, depression, anxiety, suicidal ideation, and PTSD [47,48,49,50]. Furthermore, the therapies used in these studies were consistent with those that we found in our SLR, such as cognitive-behavioral techniques [47,48,49,50], counseling [49], psychoeducation [49,50], art therapy [49,50], mindfulness and relaxation [48,49], and group interpersonal theory [48,49]. 

In terms of the empowerment components, some interventions [33,34,35,40,42,43] that included counseling, health education/psychoeducation, and expressive and cognitive therapies delivered in groups and/or individually, showed positive effects in promoting immigrant women’s empowerment. These results are presented through an increase in self-esteem, self-efficacy, general and safety-related empowerment, health literacy, and life purpose. In [43], psychoeducation and counseling resulted in significant improvements in active coping, positive reframing, self-distraction, and planning, which were significantly associated with reduced depressive symptoms. Improvements in self-esteem and self-efficacy can help immigrant women to feel more confident in their abilities and more prepared to face new challenges with positivity [51,52]. In addition, improved social problem-solving skills can help women to identify and handle problems and challenges that they may encounter through the immigration process [51]. Improvements in general and safety-related empowerment can increase women’s sense of control and security in their lives [51,53]. These findings suggest that the interventions used in the included studies can lead to positive changes in the perception that individuals have of themselves and in their ability to cope with challenges in order to find a direction and a sense of purpose in their lives, a key component of women’s empowerment [23,25], especially for those who are most vulnerable [54].

However, some studies did not find any significant results. Some reported improvements only in qualitative outcomes or found no significant differences between the experimental and control groups at pre-post-intervention or follow-up assessments. This variability can be attributed to several reasons, including differences in the quality of the interventions used, the samples studied, study designs, and outcome measures and analyses [55,56]. Additionally, three studies that assessed social support did not report significant post-intervention outcomes. Social support is a complex phenomenon and involves several stakeholders, such as community, family, friends, professionals, and others [57,58]. Therefore, preventing these variables from affecting the results becomes challenging. 

### 4.2. Effectiveness of Psychoeducational and Cognitive Restructuring Interventions to PWE of Immigrant Women

We support psychoeducation and cognitive restructuring as the strongest techniques for promoting PWE in immigrant women based on their effectiveness in the included studies, as well as their methodological quality and beyond. Psychoeducation interventions sought to provide knowledge, skills, and resources to improve immigrant women’s adaptation and well-being. Cognitive restructuring techniques were employed to help immigrant women challenge and change negative thought patterns, promoting a healthier and more adaptive perspective on themselves and their circumstances. Both interventions are accessible and easy to implement, which makes them appropriate for use in clinical and community settings. In addition, both are techniques that maintain an objectivity and standardization in their components, which allows for easier measurement, facilitating the evaluation of their effects on outcomes. Both approaches have demonstrated positive results in interventions for migrant and refugee people and their families in different contexts, such as: health treatment [59], reproductive health and mental health [60,61], detention [62], or undocumented [60] and empowerment for migrant groups [63,64]. In addition, we consulted the manual on supporting intervention for migrant women in reception centers [65] and the guide for psychological intervention with immigrants and refugees [66], which support as a main part of programs for intervention components based on techniques encompassing psychoeducation and cognitive restructuring. Finally, both approaches have found long-lasting effects in the PWE of different populations [67,68,69,70,71]. 

However, it is important to mention that none of the studies that evaluated questions about support and social problems did not have any significant results and this should be taken into consideration. This is important because the manual and the guidelines referred to the need to include the social context and the development of social skills [66]. Moreover, it is important to note that other methods and approaches were used in the studies included in this SLR, and their effects may be relevant for different populations and contexts. Consequently, the choice of interventions needs to consider the specific characteristics of immigrant women and their individual and collective needs. The toolkit “integrating migration into health interventions”, by the International Organization for Migration, highlights the importance of promoting the inclusion of migrants of all genders in multisectoral coordination mechanisms and national health bodies. This involves supporting participation in the development of action plans for migrant health and ensuring evaluation of the implementation of these plans [72]. This is relevant to promote actions that really relate to the needs of the target population. Finally, there are also barriers specific to the immigrant population that impede the implementation of health care interventions, such as language barriers, belief systems, personality, stigma, discrimination, and others [66,72,73], that need to be considered.

### 4.3. Limitations and Future Directions

This review highlighted the lack of quality of several studies [74,75,76,77,78]. There was only one randomized controlled trial, which is a more consistent design to include in the RSL and enable meta-analyses, resulting in more reliable outcomes. Furthermore, the methodological quality was low or moderate for most of the included studies, as was the presence of possible selection and confounding biases [76]. Additionally, the heterogeneity in the methods and results of the included studies made it impossible to perform a meta-analysis, restricting the synthesis of results to a descriptive and qualitative interpretation [77]. The presence of single-arm studies was a limitation in terms of interpreting the possible causal inference of the results. Furthermore, the limited availability of studies on specific interventions to improve PWE in immigrant women may have contributed to the limited number of studies included in this review. These limitations make the generalization of our results to other populations and contexts difficult [74]. To address these limitations, future studies should use more randomized controlled trials with a high methodological quality to confirm the findings and establish a strong evidence base for effective interventions. In addition, efforts should be made to standardize data collection and analysis methods to improve comparability and meta-analysis of study results. Moreover, efforts to increase the number of studies on specific interventions to improve PWE in immigrant women should be made [74,75,76,77,78].

The sample size and geographic distribution of the studies included also present some limitations. In some studies, the sample size was very small (N = 11). In addition, the total sample size of evaluated immigrant women was only 585, and 454 received some type of intervention. This is a common limitation in research with specific populations, such as immigrant women, and may affect the generalizability of the results. Small sample sizes can also increase the risk of type II errors, where a true effect is not statistically detected even if it exists in the sample. Regarding geographical issues, the studies were conducted only in the United States, Republic of Korea, and Spain and the participants were born in Latin America, Asia, Europe, and Morocco. These limitations may have affected the generalizability of the results to other populations and contexts, as the results may have been influenced by specific characteristics of each country, the small sample size, or cultural, linguistic, and contextual factors that can vary among different populations. 

Another limitation is related to the language used in the implementation of the interventions. In nine studies, there may have been problems regarding the participants’ ability to understand the intervention. This is either because the program was implemented in the language of the country (N = 5), or because the studies did not fully (N = 3) or partially (N = 1) address the language in which the program was implemented. Finally, the criteria for inclusion and exclusion of participants varied across studies, and the characteristics and needs of the participants were not homogeneous. Furthermore, the allocation of the intervention and control groups in several cases was done by convenience and in a non-equivalent format, which might have affected the individual outcomes of the participants [55,56,74]. 

For future research, it is essential to conduct studies with larger and more representative samples, including immigrant women from different backgrounds and at different stages of their migration trajectories. In addition, it is recommended to address the language of the intervention more comprehensively, ensuring cultural and linguistic adaptation, as well as the availability of translation services whenever needed. It is also important to establish clear criteria for inclusion and exclusion of participants and to enable the homogeneity of samples. The use of randomized and equivalent allocation methods between intervention and control groups is also recommended. The inclusion of interventions that address the specific needs of immigrant women in different cultural and social contexts would be noteworthy. Finally, follow-up and longitudinal studies need to be implemented to ensure that the benefits gained are maintained in the long term [74,75,76,77,78].

In addition to these limitations, the present SLR also presented some limitations regarding the inclusion or exclusion of studies that should be considered when interpreting the results. In this sense, there were possible sources of bias in our research. The restriction of the selection of publications to English, Spanish, and Portuguese languages was a limitation, which may have restricted the inclusion of relevant studies. Similarly, the use of Scopus and Web of Science databases for data collection may have ignored studies indexed in other databases. In addition, gray literature or unpublished studies were not considered, which could have contributed to a more comprehensive review. In addition, even though we adopted clear inclusion and exclusion criteria, it is possible that we excluded relevant studies due to limitations in our search strategy or interpretation of the criteria. Another limitation relates to the complex and subjective construct of psychosocial well-being and empowerment, which made objective and comparable assessments across different studies more difficult. Despite efforts to make an unbiased selection, our interpretation of these concepts may have influenced the results of the review.

To address these limitations, future studies should consider adopting a more inclusive approach to language and publication sources, including studies in additional widely spoken languages such as Chinese, Hindi, Arabic, and Bengali. Furthermore, it would be beneficial to expand the search to include other databases, like PubMed and PsycINFO, as well as considering studies from the gray literature. In terms of inclusion and exclusion criteria, future studies would benefit from enhancing the search strategy and criteria to avoid excluding relevant studies. This can be achieved through a more comprehensive literature review, adjusting selection criteria, involving additional reviewers, conducting sensitivity analyses, and being transparent about the limitations. Additionally, to address the subjective and complex nature of PWE, researchers may consider using specific and standardized assessments or including specific variables as inclusion criteria that align with the PWE construct [74,75,76,77,78]. This approach can contribute to a more comprehensive understanding of the topic and help to mitigate the limitations associated with bias and the exclusion of relevant studies. 

### 4.4. Originality and Implications

As far as we know, this is the first SLR that sought to evaluate the effects of interventions for PWE in immigrant women. Our review offers an innovative contribution by providing insights into four types of interventions (counseling, psychoeducation, cognitive therapies, expressive therapies) that have demonstrated efficacy in improving the psychological well-being of immigrant women. The results of the included studies showed significant improvements in various areas such as mood levels, depression, anxiety, stress, post-traumatic trauma, self-esteem, empowerment, and active coping skills. These findings are particularly innovative as they provide a comprehensive and holistic understanding of the effects of interventions on the psychological well-being of immigrant women, enabling the development of more comprehensive and effective interventions.

Furthermore, our findings may also contribute to the field, as we provide insights into effective and appropriate interventions for this population. Through the analysis of the included studies, we found that interventions based on psychoeducation and cognitive restructuring techniques stood out as the most effective. These innovative findings can contribute to the establishment of a solid evidence base that strengthens the clinical practice of health professionals and facilitates the development of future interventions. As these two approaches are structured and easy to implement, they can be incorporated into specific mental health and well-being programs and policies for this population, leading to positive and impactful outcomes.

Finally, our review identified gaps in the literature, which can highlight areas that require further investigation and guide future studies. The diversity of study designs and interventions highlighted in our review also emphasizes the importance of tailored approaches to address the individual needs of immigrant women. Each woman brings a unique migration experience and faces specific challenges in her adaptation process. Therefore, interventions that consider different cultural, linguistic, contextual, and socioeconomic experiences are essential to effectively address psychological well-being issues in this population. This information can guide future research, promoting a greater understanding of the most effective and appropriate interventions to improve the PWE of immigrant women and, consequently, contribute to the development of more inclusive and sensitive policies and practices tailored to their needs.

## 5. Conclusions

This SLR has provided promising evidence regarding the effectiveness of interventions aimed at improving PWE in immigrant women, despite variations in the results across studies. The experimental groups scored better in almost all variables after the interventions, even in comparison with the control group. The review also highlighted the limitations of the included studies, such as the lack of high-quality research, risk of bias, heterogeneity of methods and results, sample, and geographic locations. To address these limitations, interventions should be culturally sensitive and tailored to the specific needs of immigrant women. Furthermore, larger sample sizes and randomized controlled trials are necessary to establish the effectiveness of these interventions. These findings have important implications for health professionals and decision makers, providing guidelines for future interventions and informing health policies to consider the cultural and socioeconomic contexts of immigrant women. Finally, continued investment in research and interventions to promote PWE in immigrant women can lead to improved health and psychosocial well-being and enhance their engagement and empowerment in their new communities.

## Figures and Tables

**Table 1:** English, Portuguese, and Spanish terminology included in the search.

English	Portuguese	Spanish
Immigration	Imigração	Inmigración
Women/female	Mulheres/feminino	Mujeres/femenino
Intervention/program	Intervenção/programa	Intervención/programa
Psychosocial well-being	Bem-estar psicossocial	Bienestar psicosocial
Empowerment	Empoderamento	Empoderamiento

**Table 2:** Summary table of included studies.

N	Study ID	Study Design	Study Participants	Effect of Intervention on PWE
[33]	Bonmati-Tomas, A et al., 2019 Spain	Quasi-experiment; Pre/post-intervention; Nonrandomized; Salutogenic health; 4 sessions, 2 h, 4 weeks.	N = 28, >18 years in risk of social exclusion. 4 groups of 7 women each. Birth: Morocco, Sub-Saharan Africa, Latin American. Quantitative Tools.	Significant reduction in perceived stress, an increase in physical quality of life and a tendency towards better self-esteem. No significant improvement for sense of coherence, perceived social support, and the mental quality of life after the program.
[34]	Cho, S et al., 2012 USA	Quasi-experiment; Nonrandomized experimental and control; 4-week follow-up test logo-autobiography (LA); 6 sessions, 1 h, 6 weeks.	N = 40, >21 years and scored 16 or higher for depression. 20 in experimental group and 20 in control group. Birth: Republic of Korea. Quantitative Tools.	Experimental group reported significantly lower scores in depression and significantly higher scores in purpose in life when compared with the control group in both, posttest and at 4-week follow-up.
[35]	Choi, Y. J. 2017 Republic of Korea	Quasi-experiment; Nonrandomized; Non-equivalent experimental and control; Mental health improvement using bilingual gatekeepers; 8 sessions, 8 weeks.	N = 63, >18 years, married with a Korean citizen. 31 in experimental group and 32 in control group. Birth: Asian. Quantitative Tools.	Experimental group reported significantly better scores for mental health literacy and for the final questionnaire about text messages and scored lower for acculturative stress when compared with the control group.
[36]	Garcia, Y et al., 2020 Spain	Quasi-experiment; Non-equivalent experimental in 4 cohorts; Psychosocial therapy with text messages to mobile; 1 face-to-face session and 4 messages per day for 26 days.	N = 44, >18 years, applicants for public assistance. Birth: Latin American, European, and Morocco. Quantitative Tools.	Significant improvement in mood and depression symptoms after the intervention but only when the depression baseline was >5 (moderate depression).
[37]	Garcia, Y et al., 2019 Spain	Quasi-experiment; Nonrandomized; Non-equivalent; Experimental and control; Psychosocial therapy with text messages to mobile; 1 face-to-face session and 4 messages per day for 26 days.	N = 75, >18 years, applicants for public assistance. 46 in experimental group and 29 in control group. Birth: Latin American, European, and Morocco. Quantitative Tools.	Experimental group reported significant improvement in depression scores when compared with the control group after the intervention.
[38]	Heilemann, M. V et al., 2017 USA	Nonrandomized; Pre/post-intervention; 6-week follow-up test; Transmedia storytelling with interactive elements; 1 week of transmedia via telephone or internet.	N = 40, >21 years, and scored higher limits for depression or anxiety. Birth: Latin American. Quantitative Tools.	Significant improvement in depression and anxiety after the exposure. Evaluation after 6 weeks did not show significant results.
[39]	Jang, M et al., 2012 Republic of Korea	Quasi-experiment; Nonrandomized; Non-equivalent experimental and control; Sandplay therapy; 10 sessions, each lasting 90 min for 10 weeks.	N = 11 in an international marriage. 6 in experimental group and 5 in control group. Birth: Asian. Quantitative Tools.	Experimental group reported a significant decrease in anxiety in social interactions and in loneliness after the intervention. No significant differences between control and experimental group.
[40]	Jun, W. H et al., 2014 Republic of Korea	Quasi-experiment; Randomized; Non-equivalent experimental and control; Psychological adaptation improvement; 10 sessions, 1 or 2 sessions per week, for 8 weeks.	N = 43 in an international marriage. 21 in experimental group and 22 in control group. Birth: Asian. Quantitative Tools.	Experimental group reported a significant improvement in self-esteem and social problem solving and a lower score for depression after intervention. No significant differences between control and experimental group, only for the general social problem solving.
[41]	Kaltman, S et al., 2016 USA	Quasi-experiment; Non-equivalent experimental in 2 cohorts; A mental health intervention; 2 or 3 individual sessions and 5 sessions, 90 min each.	N = 28, >21 years, scored for depression or post-traumatic stress disorder. 4 experimental groups with 7 participants. Birth: Latin America. Quantitative and Qualitative Tools.	Significant improvement in depression and post-traumatic stress after the exposure. No significant improvement on social support after the exposure. Qualitative data presents better perception of social support.
[42]	Sabri, B et al., 2021 USA	Randomized; Pre/post-intervention; Being safe, healthy, and positively empowered; Self-directed modules, remote support over a 4-week period.	N = 70, >18 years s, and scored for depression or PTSD. Birth: Africa. Quantitative Tools.	Significant decline in stress, greater self-efficacy in trauma coping and HIV, reduced HIV/STD risk, general empowerment, and safety-related empowerment after the exposure.
[43]	Tran, A. N et al., 2012 USA	Non-randomized; Pre/post-intervention; Amigas Latinas Motivando el Alma (ALMA) focused on stress and coping; 6 sessions, 2–3 h per week, 8 weeks.	N = 48, >18 years. Birth: Latin American. Quantitative Tools.	Significant improvement in knowledge about stress management, perceived stress, depression, active coping, positive reframing, auto-distraction, humor, and planning. This knowledge was significantly associated a reduction in stress and depression.
[44]	Ko, Y et al., 2021 Republic of Korea	Experiment; Double-Blind Experimental and control; 2-week follow-up test; A laughter therapy; 4 sessions, 2 days per week, 2 weeks.	N = 41 married with a Korean. 19 in experimental group and 22 in control group. Birth: Asian. Quantitative and Physiological Tools.	Experimental group reported a significant decrease in acculturative stress, anxiety, depression, and salivary cortisol after the intervention when compared with control group. No significant differences in salivary IgA between groups.
[45]	Karasz, A et al., 2015 USA	Quasi-experiment; Randomized Experimental and control; Participatory methods to develop an asset-building mental health intervention; 12 sessions, 2 h every 15 days, 26 weeks.	N = 53, >18 years, scored 8 or higher for depression, and below the poverty level. 32 in experimental group and 21 in control group. Birth: Bangladesh. Quantitative and Qualitative Tools.	Experimental group reported a significant decrease in depression and stress after the intervention when compared with the control group. Qualitative data presents improvement in loneliness, cognitive aspects, and financial assets.

**Table 3:** JBI Appraisal Checklist for risk of bias in quasi-experimental (9 items) and experimental (13 items) studies.

N	1	2	3	4	5	6	7	8	9	10	11	12	13
[33]	X	X	X	o	X	X	X	#	X	NA	NA	NA	NA
[34]	X	X	X	X	X	X	X	X	X	NA	NA	NA	NA
[35]	X	X	X	X	X	X	X	#	o	NA	NA	NA	NA
[36]	X	X	X	o	X	X	X	#	o	NA	NA	NA	NA
[37]	X	X	X	X	X	X	X	#	o	NA	NA	NA	NA
[38]	X	X	X	o	X	X	X	#	o	NA	NA	NA	NA
[39]	X	X	X	X	X	X	X	X	o	NA	NA	NA	NA
[40]	X	X	X	X	X	X	X	X	X	NA	NA	NA	NA
[41]	X	X	X	o	X	X	#	#	o	NA	NA	NA	NA
[42]	X	X	X	o	X	X	X	#	X	NA	NA	NA	NA
[43]	X	X	X	o	X	X	X	o	X	NA	NA	NA	NA
[44]	X	X	X	#	X	#	X	X	X	X	X	#	X
[45]	X	X	X	X	X	X	X	#	o	NA	NA	NA	NA

Met requirement (X), didn’t meet requirement (o), confused (#), not applicable (NA).

## Data Availability

To request the dataset extracted for this SLR, send an email to Patricia Silva at pg.silva@ubi.pt. The data will be provided for academic or research purposes, and only if the ethics and privacy policies of both the institution and the research project are respected.

## Appendix A. Checklist PRISMA 2020 for Abstracts

This appendix provides the checklist for reporting systematic reviews and meta-analyses in abstracts following the PRISMA 2020 guidelines. The checklist includes essential information that should be included in the abstract to ensure transparency and completeness in reporting.

**SELECTION** **AND TOPIC**	**ITEM**	**CHECKLIST ITEM**	**REPORTED (YES/NO)**
**TITLE**			
Title	1	Identify the report as a systematic review.	YES
**BACKGROUND**			
Objectives	2	Provide an explicit statement of the main objective(s) or question(s) the review addresses.	YES
**METHODS**			
Eligibility Criteria	3	Specify the inclusion and exclusion criteria for the review.	YES
Information Sources	4	Specify the information sources (e.g., databases, registers) used to identify studies and the date when each was last searched.	YES
Risk of Bias	5	Specify the methods used to assess risk of bias in the included studies.	YES
Synthesis of Results	6	Specify the methods used to present and synthesize results.	YES
**RESULTS**			
Included Studies	7	Give the total number of included studies and participants and summarize relevant characteristics of studies.	YES
Synthesis of Results	8	Present results for main outcomes, preferably indicating the number of included studies and participants for each.	YES
**DISCUSSION**			
Limitations of Evidence	9	Provide a brief summary of the limitations of the evidence included in the review (e.g., study risk of bias, inconsistency and imprecision).	YES
Interpretation	10	Provide a general interpretation of the results and important implications.	YES
**OTHER**			
Funding	11	Specify the primary source of funding for the review.	YES
Registration	12	Provide the register name and registration number.	YES

## Appendix B. Checklist PRISMA for Systematic Review

Appendix B provides the checklist for reporting a systematic review according to the PRISMA 2020 guidelines. It includes essential items to ensure the transparency and integrity of the SLR, such as title, abstract, introduction, methods, results, discussion, and other topics such as protocol registration, funding, conflicts of interest, and data availability. The second column shows the lines in which each topic can be found in our text.

**SELECTION** **AND TOPIC**	**ITEM**	**CHECKLIST ITEM**	**REPORTED (YES/NO)**
**TITLE**			
Title	1	Identify the report as a systematic review.	3
**ABSTRACT**			
Abstract	2	Provide an explicit statement of the main objective(s) or question(s) the review addresses.	11–36
**INTRODUCTION**			
Rationale	3	Describe the rationale for the review in the context of existing knowledge.	79–82
Objectives	4	Provide an explicit statement of the objective(s) or question(s) the review addresses.	83–86
**METHODS**			
Eligibility Criteria	5	Specify the inclusion and exclusion criteria for the review and how studies were grouped for the syntheses.	126–156
Information Sources	6	Specify all databases, registers, websites, organisations, reference lists, and other sources searched or consulted to identify studies. Specify the date when each source was last searched or consulted.	92–106
Search Strategy	7	Present the full search strategies for all databases, registers, and websites, including any filters and limits used.	107–125
Selection Process	8	Specify the methods used to decide whether a study met the inclusion criteria of the review, including how many reviewers screened each record and each report retrieved, whether they worked independently, and if applicable, details of automation tools used in the process.	157–163 329–335
Data Collection Process	9	Specify the methods used to collect data from reports, including how many reviewers collected data from each report, whether they worked independently, any processes for obtaining or confirming data from study investigators, and if applicable, details of automation tools used in the process.	N/A
Data Items	10a	List and define all outcomes for which data were sought. Specify whether all results that were compatible with each outcome domain in each study were sought (e.g., for all measures, time points, analyses) and, if not, the methods used to decide which results to collect.	308–319
	10b	List and define all other variables for which data were sought (e.g., participant and intervention characteristics, funding sources). Describe any assumptions made about any missing or unclear information.	200–204
Study Risk of Bias Assessment	11	Specify the methods used to assess risk of bias in the included studies, including details of the tool(s) used, how many reviewers assessed each study and whether they worked independently and, if applicable, details of automation tools used in the process.	265–274 329–335
Effect Measures	12	Specify for each outcome the effect measure(s) (e.g., risk ratio, mean difference) used in the synthesis or presentation of results.	NA
Synthesis Methods	13a	Describe the processes used to decide which studies were eligible for each synthesis (e.g., tabulating the study intervention characteristics and comparing against the planned groups for each synthesis (item #5).	311–317
	13b	Describe any methods required to prepare the data for presentation or synthesis, such as handling of missing summary statistics, or data conversions.	311–317
	13c	Describe any methods used to tabulate or visually display results of individual studies and syntheses.	329
	13d	Describe any methods used to synthesize results and provide a rationale for the choice(s). If meta-analysis was performed, describe the model(s), method(s) to identify the presence and extent of statistical heterogeneity, and software package(s) used.	320–328
	13e	Describe any methods used to explore possible causes of heterogeneity among study results (e.g., subgroup analysis, meta-regression).	NA
	13f	Describe any sensitivity analyses conducted to assess robustness of the synthesized results.	NA
Reporting Bias Assessment	14	Describe any methods used to assess risk of bias due to missing results in a synthesis (arising from reporting biases).	NA
Certainty Assessment	15	Describe any methods used to assess certainty (or confidence) in the body of evidence for an outcome.	298–304
**RESULTS**			
Study Selection	16a	Describe the results of the search and selection process, from the number of records identified in the search to the number of studies included in the review, ideally using a flow diagram.	163–199
Synthesis of Results	16b	Cite studies that might appear to meet the inclusion criteria, but which were excluded, and explain why they were excluded.	174–179
Study Characteristics	17	Cite each included study and present its characteristics.	Table 1 208–264
Risk of Bias in Studies	18	Present assessments of risk of bias for each included study.	274–283 Table 2
Results of Individual Studies	19	For all outcomes, present, for each study: (a) summary statistics for each group (where appropriate), and (b) an effect estimate and its precision (e.g., confidence/credible interval), ideally using structured tables or plots.	337–414
Results of Syntheses	20a	For each synthesis, briefly summarize the characteristics and risk of bias among contributing studies.	362–363 377–379 400–401 417–418
	20b	Present results of all statistical syntheses conducted. If meta-analysis was done, present for each the summary estimate and its precision (e.g., confidence/credible interval) and measures of statistical heterogeneity. If comparing groups, describe the direction of the effect.	337–414
	20c	Present results of all investigations of possible causes of heterogeneity among study results.	NA
	20d	Present results of all sensitivity analyses conducted to assess the robustness of the synthesized results.	NA
Reporting Biases	21	Present assessments of risk of bias due to missing results (arising from reporting biases) for each synthesis assessed.	N/A
Certainty of Evidence	22	Present assessments of certainty (or confidence) in the body of evidence for each outcome assessed.	361–362 375–377 399–400 416–417
**DISCUSSION**			
Discussion	23a	Provide a general interpretation of the results in the context of other evidence.	424–501
	23b	Discuss any limitations of the evidence included in the review.	502–552
	23c	Discuss any limitations of the review processes used.	553–580
	23d	Discuss implications of the results for practice, policy, and future research.	581–611
**OTHER**			
Registration and Protocol	24a	Provide registration information for the review, including register name and registration number, or state that the review was not registered.	633–634
	24b	Indicate where the review protocol can be accessed, or state that a protocol was not prepared.	634–635
	24c	Describe and explain any amendments to information provided at registration or in the protocol.	636–643
Support	25	Describe sources of financial or non-financial support for the review, and the role of the funders or sponsors in the review.	644–645 652–654
Competing Interests	26	Declare any competing interests of review authors.	652
Availability of data, Code, and Other Materials	27	Report which of the following are publicly available and where they can be found: template data collection forms; data extracted from included studies; data used for all analyses; analytic code; any other materials used in the review.	649–651

## Appendix C. Search Strategy in the Databases

This appendix outlines the search strategy used to retrieve relevant studies in the databases for our systematic review. The strategy was developed for the Web of Science and Scopus databases, in the English, Portuguese, and Spanish languages. Additionally, some common criteria for both databases are presented.

**Database**	**Language**	**Search Strategy**
Web of Science	English	(ts=(intervent*)) or ts=(program) and ts=(*mmigra*) and (ts=(*wom*)) or ts=(female) and ((ts=(empower*)) or ts=(psych*)) or all=(well*)
	Portuguese	(ts=(intervenç*)) or ts=(programa*) and ts=(*migra*) and (ts=(mulher*)) or ts=(feminin*) and ((ts=(empodera*)) or ts=(psico*)) or ts=(bem*)
	Spanish	(ts=(intervención*)) or ts=(programa*) and ts=(*inmigra*) and (ts=(mujer*)) or ts=(femenin*) and ((ts=(empodera*)) or ts=(psico*)) or ts=(bienestar*)
Scopus	English	(title-abs-key (“wom*” or “female”) and title-abs-key (“*mmigra*”) and title-abs-key (“intervent*” or “program*”) and title-abs-key (“psych*” or “empower*” or “well*”))
	Portuguese	(title-abs-key (“mulher*” or “feminin*”) and title-abs-key (“*migra*”) and title-abs-key (“intervenç**” or “programa*”) and title-abs-key (“psico” or “empodera*” or “bem*”))
	Spanish	(title-abs-key (“mujer*” or “femenin*”) and title-abs-key (“*inmigra*”) and title-abs-key (“intervencion**” or “programa*”) and title-abs-key (“ppsico” or “empodera*” or “bienestar*”)
Common Criteria	YEAR OF PUBLICATION = 2010–2023 LANGUAGE = Portuguese, English, and Spanish TYPE OF THE STUDY = published paper, conference paper, editorial content * = The asterisk represents a sequence of characters that are unknown or that can be substituted or supplemented	

## Appendix D. Summary Table with Description of the Studies’ Interventions Plan

Appendix D presents a summary that provides information about the intervention plan adopted in the studies included in the systematic review. The table contains details about the type of intervention plan, the program language used, and a brief description of the intervention plan in each study. In our SLR the studies were constructed based on sessions, contents, or implementation phases.

**N**	**Type** **of Plan**	**Language** **of the Program**	**Description of Intervention Plan**
[33]	SESSIONS	Does not address.	SESSION 1: Self-knowledge of personal generalized resistance resources (GRR). SESSION 2: Identifying community GRRs, family role, and social support. SESSION 3: Exploring the role of each participant in her community. SESSION 4: Building individual capacity to complete a personal project.
[34]	SESSIONS	Native language of the participants.	SESSION 1: Self-introduction, orientation, and production of a chronology. SESSION 2: Significant relationships in one’s life before migration. SESSION 3: Exploring relationships in one’s life after migration. SESSION 4: Exploring crises and self-attitude towards crises before migration. SESSION 5: Exploring crises and self-attitude towards crises after migration.SESSION 6: Reading someone’s autobiography.
[35]	SESSIONS	Native language of the participants.	SESSION 1: Acculturative stress. SESSION 2: Acculturative stress management. SESSION 3: Satisfactory relationships—husband. SESSION 4: Joyful relationships—children. SESSION 5: Harmonious relationships with the family-in-law. SESSION 6: Mental health literacy. SESSION 7: Mental health management.SESSION 8: Utilizing mental health services.
[36]	CONTENTS	Native language of most of the participants.For the others, it is not stated.	Contents included a message bank focused in: (a) strengthening of positive thinking, (b) capacity for reflection, (c) improvement of health, (d) promotion of physical activity, (e) capacity for planning and organization of tasks, and (f) improvement of social relations.
[37]	CONTENTS	Language of the country.The participants were able to communicate in that language.	Contents included a message bank focused on: (1) thinking, (2) health, (3) physical activity, and (4) social relationships. The messages inquired, advised, and guided regarding daily life habits to develop positive and healthy behaviors as opposed to the negative.
[38]	CONTENTS	Language of the country.The participants were able to communicate in that language.	Contents included photos, captions, and links to all videos and the resource-rich blog, including contact information and links to local and web-based resources, as well as hotlines to mental health services.
[39]	SESSIONS	Does not address.	All sessions included each participant selecting five photos, doing a sandbox scene in silence, and recounting the emotions, physical sensations, memories, and ideas they felt while doing the sandbox scene. Then, two participants were put in pairs and one of them did a sand scene while the other became an observer of the work and reported back to the group. In the end everyone shared with the group.
[40]	PHASES	Language of the country.The participants were able to communicate in that language.	PHASE 1: Cognitive reconstruction, producing a positive self-image and developing your own strengths, understanding emotions and behaviors caused by cognitive distortion, and reinforcing rational thoughts. PHASE 2: Strengthening problem solving skills led participants to change their problem coping behavior through problem solving skill development. PHASE 3: Practice of integrating the contents learned through dramatization.
[41]	CONTENTS	Native language of the participants.	The content included behavioral activation (BA), a component of CBT for depression and PTSD. BA seeks to find problem behaviors and idleness and implements programs to help individuals become more active in their natural environment by connecting them to sources of reinforcement. It includes monitoring and evaluation of the enjoyment gained from participation in activities. Gradual attributions to involvement in enjoyable activities. Cognitive testing of programmed activities for possible obstacles.
[42]	SESSIONS	Language of the country.The participants were able to communicate in that language.	SESSION 1: Individual strengths-based telephone feedback based on survey responses and initial assessment. SUBSEQUENT SESSIONS: Self-directed online educational/psychoeducational modules and stress-reduction phone sessions facilitated by the research team. They addressed topics such as sexual-reproductive health, healthy relationships, immigration, HIV/STDs, career planning and financial management, family planning, and information on immigration visas or local service providers.
[43]	SESSIONS	Native language of the participants.	FIRST FIVE SESSIONS: Migration Journey; Life in NC and You: Ways of Adjusting to a New Place; Mental health; Helping Others as a Promoter; and Life in NC and Family: Difficulties Adjusting to a New Location. FINAL SESSION: Included a graduation event with family and peers.
[44]	SESSIONS	Language of the country.The participants were able to communicate in that language. There was a translator if necessary.	Contents included various activities along with simulated laughter such as clapping, singing, dancing, and playing.
[45]	PHASES	Does not address.	PHASE 1: Develop psychological skills through cognitive strategies for identifying tension symptoms and the thoughts and feelings associated with them. Psychoeducation on mental health. PHASE 2: Strengthen social networks and friendships among women. Involve partners. PHASE 3: Construction of financial assets. Basic financial education and household budget information. Opening a US$10 savings account to acquire assets that can contribute to financial independence.

## Appendix E. Summary of Data Collection and Analysis Instruments of the Studies

In this appendix, we present a summary of the data collection and analysis instruments used by the studies that were included in the systematic review. The data collection instruments included structured questionnaires, semi-structured interviews, and physiological analyses. The data analysis uses quantitative and qualitative methods.

**N**	**Data Collection**	**Data Analysis**
[33]	Quantitative: Sense of coherence (SOC-13); Perceived social support (Duke-UNC-11); Quality of Life, QoL Short Form-36 (SF-36); Rosenberg’s Self-Esteem Scale; Perceived Stress Scale (PSS-10).	T-test; Wilcoxon test; Pearson’s correlation; Kolmogorov–Smirnov; Shapiro–Wilk tests; Multiple regression.
[34]	Quantitative: Center for Epidemiologic Studies Depression (CES-D); Purpose in Life (PIL).	ANOVA; Mann–Whitney, U test.
[35]	Quantitative: The Mental Health Literacy Inventory (MHL); The Acculturative Stress Scale; The Korean General Health Questionnaire (KGHQ).	ANCOVAs.
[36]	Quantitative: Final Questionnaire on Text Messages (FQTM); Patient Health Questionnaire (PHQ-9).	T-test; Wilcoxon test.
[37]	Quantitative: Final Questionnaire on Text Messages (FQTM); Patient Health Questionnaire (PHQ-9).	T-test.
[38]	Quantitative: Patient Health Questionnaire; (PHQ-9); Brief Generalized Anxiety Disorder Scale (GAD-7).	Spearman correlation test.
[39]	Quantitative: Social Interaction Anxiety Scale; UCLA loneliness scale.	Wilcoxon test; Mann–Whitney U test.
[40]	Quantitative: Self-Esteem Scale (SES); Center for Epidemiological Studies Depression Scale (CES-D); Revised Social Problem-Solving Inventory (SPSI-R).	T-test; Fisher’s exact test; ANOVA.
[41]	Quantitative: Stressful Life Events Screening Questionnaire (SLESQ); PTSD Checklist; Patient Health Questionnaire-9 (PHQ-9); Medical Outcomes Study Social Support (MOS-SSS).Qualitative: Semi-structured interview.	T-test; Content analysis.
[42]	Quantitative: Perceived Stress Scale; Inventory of Positive Psychological Attitudes; Trauma Coping Self-Efficacy Scale; Revised cognitive and affective mindfulness scale; Safe sexual behavior questionnaire; HIV Knowledge Questionnaire; STD Knowledge Questionnaire; Self-Efficacy Scale for STI-HIV; Revised Personal Progress Scale; Safety Related Victim; Empowerment Measure (MOVERS); Harvard Trauma Questionnaire; Patient Health Questionnaire.	T-test; Cohen’s d;
[43]	Quantitative: Scale of 16 statements to assess the role of the Promoter; Perceived Stress Scale (PSS); Center for Epidemiological Studies Depression Scale (CES-D); Multidimensional Scale of Perceived Social Support; Short version of Brief COPE.	T-test; Beta coefficient.
[44]	Quantitative: The acculturative stress index; The Center for Epidemiological Studies-Depression Scale (CES-D); The Beck Anxiety Inventory. Physiological analysis: Salivary cortisol and salivary; IgA levels.	Fisher’s exact test; T-test; Post hoc; ANOVA.
[45]	Quantitative: Patient Health Questionnaire-9 (PHQ-9); The South Asian Stress Scale. Qualitative: Semi-structured interview.	Analysis of variance; Qualitative analysis of coding and categorization.
